# Phytoplankton Sensitivity to Heavy Metals in Baltic Coastal Lakes

**DOI:** 10.3390/ijerph19074131

**Published:** 2022-03-31

**Authors:** Monika Szymańska-Walkiewicz, Katarzyna Glińska-Lewczuk, Paweł Burandt, Krystian Obolewski

**Affiliations:** 1 Department of Hydrobiology, University of Kazimierz Wielki in Bydgoszcz, 85-090 Bydgoszcz, Poland; obolewsk@ukw.edu.pl; 2 Department of Water Resources and Climatology, University of Warmia and Mazury in Olsztyn, 10-719 Olsztyn, Poland; kaga@uwm.edu.pl (K.G.-L.); pawel.burandt@uwm.edu.pl (P.B.)

**Keywords:** phytoplankton, coastal lakes, heavy metals, surface water

## Abstract

This study aimed to compare concentrations of chlorophyll-a between individual phytoplankton groups for four shallow Baltic coastal lakes, varying in type of connection with the sea. For two years, the research focused on quantifying the effects of abiotic factors—concentrations of heavy metals (Ba, Bi, Cr, Cu, Mn, Fe, Ni, Pb, and Zn) and hydrological connectivity—on phytoplankton composition, biomass, and photosynthetic activity. Our results show that hydrological factors are the main predictors of phytoplankton structure. The lakes differed in salinity: freshwater vs. brackish vs. transitional lakes. Irrespective of lake type, the dominant group was that of Cyanobacteria (~80%), but their percentage contribution was lower in the brackish lake. Baltic seawater intrusion resulted in a decrease in heavy-metal concentrations in lake water for Fe, Zn, Pb, and Bi. Redundancy analysis (RDA) suggested positive effects of some heavy metals on the biomass of the Chlorophyta and Bacillariophyta. For the Cryptophyta only, a slight decrease in biomass was linked with increased metal concentrations in open water.

## 1. Introduction

Coastal lakes represent a specific type of aquatic ecosystem at the interface between land and sea, in which complex physical, chemical, and biological processes take place that have an important function in the biogeochemical cycle of metals. The influence of metals on physiological processes between heavy metals and plankton organisms is one of the keys to explaining the role of trace elements in coastal lake function, including bioaccumulation and biomagnification processes [1].

Coastal ecosystems share characteristic variations in hydrodynamics and physicochemical water property dynamics caused by terrestrial and/or marine factors [2]. The terrestrial influence is determined mostly by a lake catchment’s characteristics and its water supply, while the marine influence depends primarily on the degree of hydrological connectivity with the sea. Anthropogenic activity in a lake catchment is widely known to be a key factor in degrading the trophic conditions of a water body. The development of the primary productivity and biomass of phytoplankton, particularly Cyanobacteria, is stimulated by an excess of nutrients and pollutants, which can be recharged both from the catchment and from internal sources (secondary pollution) [3,4]. This is also the case for Baltic coastal lakes, where the threat of overfertilization and water pollution is still a severe problem.

The susceptibility of a coastal lake to deterioration or degradation relates to hydrological connectivity. Coastal water bodies can be classified as one of three major types of aquatic ecosystems: open lagoons (where the intrusion of seawater prevails); intermittently open and closed lagoons (with limited intrusion); permanently closed, isolated lakes [5]. However, coastal lakes of brackish seas (e.g., the Baltic Sea) can also be classified by salinity into freshwater, transitional (brackish-freshwater and freshwater-brackish), and brackish [6,7]. Among them, transitional lakes show the most distinct instability of abiotic conditions. Lakes of this type are very difficult to classify; depending on the amount and intensity of the periods of connectivity with sea waters, they become more similar to freshwater or brackish lakes. Hence, the need to introduce the terms “brackish-freshwater” and “freshwater-brackish” lakes to facilitate further analysis.

Since they are located close to river mouths, coastal aquatic ecosystems are more exposed to pollution and loaded with heavy metals than other aquatic ecosystems [8,9,10]. Coastal waters, especially brackish ones, act as transition zones where weathering material from the catchment is trapped and through which some of the material is transported to the open sea. In the case of lakes along the southern Baltic Sea coast, their pollution loads are primarily of agricultural origin [10,11] or derive from domestic sewage discharged both directly from land and indirectly, through seawater intrusion, from the sea. Moreover, coastal lakes, due to their shallow depths, are particularly susceptible to wind-induced water mixing and resuspension of the mineral and organic matter stored in bottom sediments. Under these conditions, heavy metals and other chemical elements are released from sediments and lead to the increase of suspended solids (TSS), as well as the secondary contamination of water. As a consequence, the metals in water undergo an array of biogeochemical processes that control the solubility, mobility, bioavailability, and toxicity of metals in the environment [12]. Thus, in comparison to inland freshwater environments, coastal lakes may have a more severe ecological imbalance and deterioration in physicochemical water properties, which affects aquatic biodiversity [13]. Contamination with heavy metals can negatively affect aquatic organisms (both freshwater and marine) at the cellular level, causing oxidative stress. This effect is particularly conspicuous in the case of phytoplankton, as confirmed by many ecotoxicological studies [14,15,16]. Research has shown that metals in water can strongly affect the productivity and chemical composition of phytoplankton [17]. Each algal group can respond differently depending on the types and concentrations of elements acting upon them [18]. Considering their short life cycles and relatively high sensitivity to changes in the environment, phytoplankton are thus ideal model organisms for studies of land–water interactions and may be used to identify and describe physiological, biochemical, and ecological processes in transitional zones between land and sea [19,20].

At the interface between the land and sea, it is often difficult to interpret the complexity of sediment–water interactions, which are related to changes in the balance of metal adsorption and desorption along the salinity gradient, and to physical processes such as river flow, tidal energy, and currents. Still, little is known about how either the response of algal populations to the presence of metals in the turbid waters of coastal lakes or hydrological connectivity with the sea influence their behavior. Heavy metals with potential relevance to the producer activity were selected from the literature [21].

The major goal of this study was to investigate relations between heavy metal concentrations (Ba, Bi, Cr, Cu, Mn, Fe, Ni, Pb, and Zn) and the abundance of phytoplankton (chlorophyll-a content of individual groups) and its photosynthetic activity in four shallow Baltic coastal lakes that vary in the type and degree of connection with the sea.

## 2. Materials and Methods

### 2.1. Study Area

**Figure 1:**
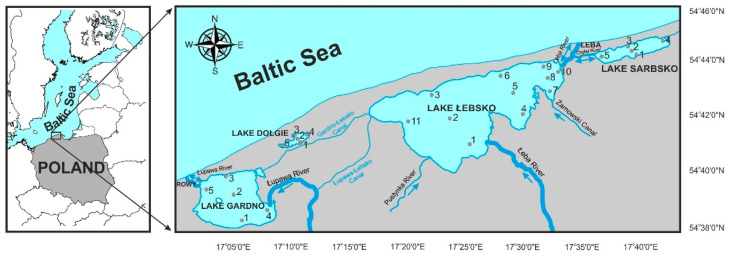
Locations of the studied coastal lakes of the southern Baltic Sea and sampling sites (numbers).

The lakes included in this study are located within, or in the buffer zone of, the Słowiński National Park (SNP) (54°42′22″ N, 17°17′55″ E), which is of exceptionally high ecological value. The area is protected by UNESCO’s Man and Biosphere Programme, within the World Network of Biosphere Reserves, and the Ramsar Convention on Wetlands of International Importance. Freshwater habitats in the SNP cover 30% of the area, mostly represented by three polymictic lakes: Łebsko, Gardno, and Dołgie. Additionally, Lake Sarbsko is located in the eastern part of the buffer zone of the SNP (Figure 1). All of them are interlinked by a system of canals or share a connection to the Baltic Sea. Thus, they form an interconnected, extensive hydrological system.

The lakes can be divided into four types depending on their hydrological connectivity with the sea. The classification of lakes in this study was based on salinity according to Dam et al. [22]. We revealed remarkable differences in salinity level between the lakes, so the terms brackish (B), transitional, and freshwater (F) were adopted. In transitional lakes, the possibility of sea water intrusion made it necessary to subdivide them into brackish-freshwater (BF) and freshwater-brackish (FB) as a function of residence time according to Obolewski et al. [7] (Table 1).

Lake Łebsko is the largest shallow lake on the Polish coast, with the water table area of 7020 ha (Table 1). Its main tributary is the Łeba River. Its total watershed area amounts to 1801.2 km^2^. The river supplies the lake with a mean flow rate of 12.73 m^3^·s^−1^, while the river flow at the river mouth into the sea amounts, on average, to 18.81 m^3^·s^−1^. The river channel allows the free long-term intrusion of seawater, particularly during landward winds, so the lake is brackish (B). The major tributary of Lake Gardno is the Łupawa River, which enters the lake with a mean flow rate of 8.3 m^3^·s^−1^. The connection with the sea assures a 9.8-km-long canal, which allows the frequent intrusion of seawater. Gardno is linked with Łebsko through the Gardno-Łebsko Canal. A similar bypass links the Łupawa River with Lake Łebsko. As a result, Gardno is classified as a transitional coastal lake with a dominance of brackish water over freshwater (FB). Lake Sarbsko is fed by the Chełst River, which is connected with the most downstream section of the Łeba River and Baltic Sea. This interconnection allows for the periodical intrusion of seawater, raising salinity in the lake. Lake Sarbsko is classified as transitional, but with a dominance of freshwater over brackish water (BF), in contrast to Lake Gardno. The freshwater Lake Dołgie (F) is a small water body, completely isolated from the sea. It is supplied only sporadically with freshwater by the Gardno–Łebsko Canal (Table 1).

### 2.2. Water Sampling

The study was conducted in 2017–2018 in four coastal lakes (Figure 1) representing distinct hydrological types: Łebsko (brackish, 11 sampling sites), Gardno (freshwater-brackish, 5 sites), Sarbsko (brackish-freshwater with an occasional inflow of seawater, 5 sites), and Dołgie (freshwater, 5 sites). In total, 156 samples were taken: 66 from Lake Łebsko and 30 each from the smaller lakes. At each sampling site, water samples were collected in containers (2.5 L) with the use of a Patalas sampler from a depth of 0.5–1.0 m.

### 2.3. Laboratory Procedure

Within 24 h of collection, water samples were analyzed in the laboratory. To assess metal concentrations in water, water samples were evaporated to 20–25 cm^3^ and then mineralized. For preparation of the reference materials and samples, we used 65% HNO_3_ (Merck, Darmstadt, Germany) and 30% hydrogen peroxide (Sigma Aldrich, Darmstadt, Germany). To measure concentrations of selected metals that can potentially affect phytoplankton structure (Ba, Bi, Cr, Cu, Fe, Mn, Ni, Pb, and Zn), we used an Agilent 5100 inductively coupled plasma optical emission spectrometer (ICP-OES) (Agilent, Santa Clara, CA, USA). The chemical analysis was repeated three times to obtain reliable results with minimized measurement errors. The results were averaged only if the differences between the three results were lower than 5%. For ICP-OES, we used commercial analytical inductively coupled plasma (ICP) standards (Romil Ltd., Cambridge, UK). Detection limits, determined as three-criteria, were estimated at 0.001 mg·L^−1^ and 0.01 mg·kg^−1^ dry weight (DW) for all the studied elements. The uncertainty of the whole analytical procedure (including sample preparation) reached 20% [24].

The water samples for phytoplankton analyses were kept in darkness until analysis (~4 h) to obtain the optimal fluorescence intensity. In the laboratory, biological material was poured into a glass 25-mL cuvette and analyzed using a spectral AlgaeLabAnalyse (ALA) fluorimeter (BBE, Schwentinental, Germany). The fluorimeter measured chlorophyll-a fluorescence at 685 nm following excitation of the photosynthetic pigments by light-emitting diodes at five different wavelengths (450, 525, 570, 590, and 610 nm; for a detailed description, see Beutler et al. [25,26]. Total chlorophyll-a (TChl, μg·L^−1^) and phytoplankton community composition were measured also using the ALA. Excitation spectra were compared against a library of signature spectra (“fingerprints”) for four algal groups: Bacillariophyta, Chlorophyta, Cyanobacteria, and Cryptophyta. Each measurement record was the arithmetic mean of three subsamples. We used total chlorophyll-a as a measure of biomass and the abundance of spectrally-identified algal groups, as well as photosynthetic activity (Genty method, %) [27]. For proper calculation of the total chlorophyll-a concentration, we corrected for yellow substances using the chromophoric dissolved organic matter (CDOM).

### 2.4. Statistical Analysis

To limit the influence of absolute values, the biomass of individual groups of phytoplankton was square-root transformed (√ (x + 1)), while environmental data were log-transformed (log_10_ (x + 1)) [28]. To assess environmental conditions, the following parameters were used: concentrations of heavy metals in lake water, concentrations of chlorophyll-a in four groups of phytoplankton (Chlorophyta, Bacillariophyta, Cyanobacteria, and Cryptophyta) and photosynthetic. We performed an analysis of variance (ANOVA) using the Kruskal–Wallis test (K–W), followed by the post-hoc Dunn test (*p* < 0.05) in GraphPadPrism 5.01 software (GraphPad, San Diego, CA, USA). The residuals were tested for normality (Shapiro–Wilk test) and homoscedasticity (Levene test).

Coefficients of correlation (r) between individual values for each measure were determined using the Spearman test. Next, we used the Draftsman technique of scatter plot displays. Scatter plots of heavy metals versus phytoplankton were created to examine associations.

In the next stage, we used the linear model of redundancy analysis (RDA) to explain the biomass of the studied groups of phytoplankton and to associate them with environmental variables (including metal concentrations in water) in individual lake types. We used the Monte Carlo technique with 999 permutations. T-value biplots with van Dobben circles were generated, based on the RDA of physicochemical properties of water (individual heavy metal) and algal groups to illustrate statistically significant relationships between the organisms and environmental variables [29].

For heavy metals, we first generated generalized linear models (GLM, *p* < 0.05), followed by RDA models [30]. The models were run using the statistical software Canoco 5.0 (New York, NY, USA).

## 3. Results

### 3.1. Heavy Metals

Mean concentrations decreased in the following order: Fe > Zn >Mn > Pb > Ni > Ba > Cr > Cu > Bi. Among the heavy metals in coastal lakes, 60% differed significantly between lake types. The concentrations of Ba, Bi, Cr, and Cu between BF and FB waters were the only ones that were similar. At the same time, concentrations of Mn declined with decreasing lake water exchange with the sea, as did Cu and Ba to a lesser extent (Table 2). The highest metal concentration was that of Fe in the freshwater lake (F); it made the total concentration of heavy metals significantly higher in F than in the other lake types (Dunn test, *p* < 0.0001).

Significant seasonal changes in concentrations of the metals were detected among lakes, particularly the lakes of types F and B. The highest variability for Cr, Fe, and Mn was found in the lakes of types F and B, and for Cu in type B. In lakes of transitional character (BF type), significant variations of Cu and Ni, as well as Fe and Mn, were found. There were significant seasonal variations in the concentrations of the studied metals in all seasons, as follows: Ba (*p* < 0.0001), Bi (*p* < 0.0006), and Fe (*p* < 0.0002). Concentrations of Cu (*p* = 0.0003) and Zn (*p* = 0.03) differed statistically in the summer, while Mn (*p* < 0.0001) differed in the spring. Data on the concentrations of metals in lake water in each season are given in the supplementary material (Table S1).

### 3.2. Phytoplankton Biomass Structure

Chlorophyll-a concentration, as a measure of phytoplankton biomass, differed significantly between individual lake types (Table 3). The difference was due primarily to the chlorophyll-a of the Cryptophyta, which was significantly higher in BF than in the other lake types (Dunn test, *p* = 0.0001). Substantial differences between lakes also applied to the biomass of the Bacillariophyta, especially for F vs. B (*p* = 0.0007), BF (*p* = 0.0005), and also FB (*p* = 0.03). However, irrespective of the level and type of connectivity with the sea, the Cyanobacteria were a major component of phytoplankton in the coastal lakes. Despite this, the biomass of this group of phytoplankton differed significantly between F and B (*p* = 0.002). Cyanobacteria were the most abundant in BF (where they accounted for 82% of total chlorophyll-a) and in T (86%). Chlorophyll-a concentrations of the Chlorophyta were significantly higher in F than in BF (*p* < 0.003), B (*p* = 0.003), and FB (*p* = 0.01). Seasonal changes in phytoplankton parameters were most distinct in the lakes of type F. The differences were found to be of significant importance for diatom biomass (*p* < 0.04), cryptophytes (*p* = 0.01), and PA (*p* < 0.002). Photosynthetic activity also underwent strong seasonal variations in lakes F (*p* < 0.0001) and FB (*p* < 0.03). In the lakes of type BF, the biomass of greens (*p* < 0.0001) and diatoms (*p* < 0.03) were characterized by strong seasonal variation. Only in the B type of lakes was a seasonal change in CPOM recorded (*p* = 0.02). Significant differences in biomass were recorded in greens (*p* < 0.003), diatoms (*p* < 0.02), and cryptophytes (*p* = 0.0003) in spring, in greens (*p* = 0.01), Cyanobacteria (*p* < 0.02) and TCHL (*p* < 0.03) in summer and in diatoms (*p* = 0.005), cryptophytes (*p* = 0.009) and TCHL (*p* < 0.03) in autumn. Data on chlorophyll-a concentrations in the lake waters and seasonal change analysis results are given in the Supplementary Materials (Table S2).

### 3.3. Variables Affecting Concentrations of Phytoplankton Groups

**Figure 2:**
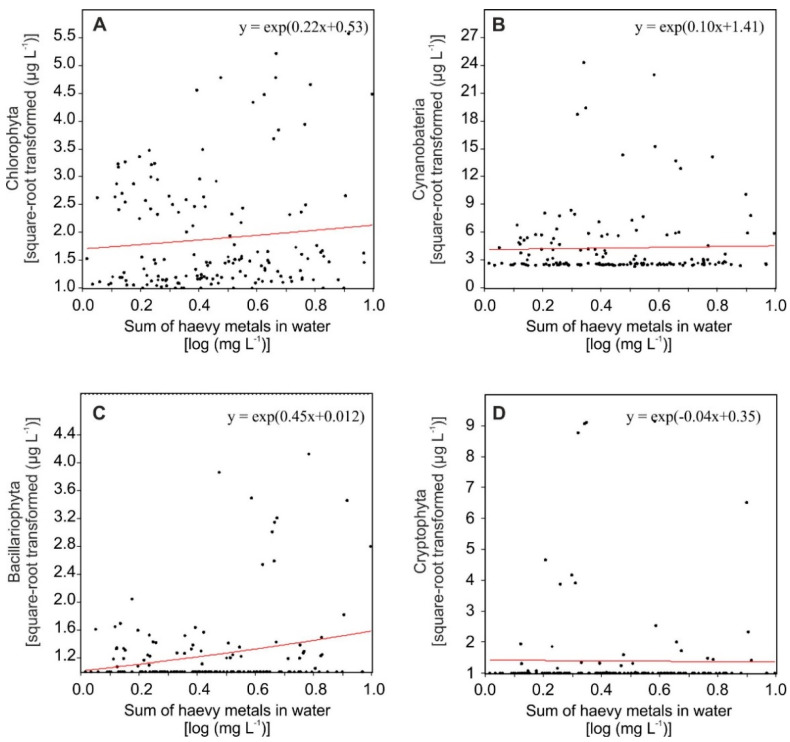
Generalized linear model for phytoplankton group metrics as response variables, and the sum of heavy metals as an explanatory variable, with the link function (log) based on chlorophyll-a concentration, as a measure of the biomass of the Chlorophyta (**A**), Cyanobacteria (**B**), Bacillariophyta (**C**), and Cryptophyta (**D**).

The analysis of GLM showed that the concentrations of the nine heavy metals in surface waters of the coastal lakes were positively related to the biomass of the Chlorophyta and Bacillariophyta (Figure 2A,C), but they did not seem to be associated with the Cyanobacteria (Figure 2B). For Cryptophyta, only a slight decrease in biomass was linked with increased metal concentrations in coastal lake water (Figure 2D).

We used the results of this model to generate a matrix of Spearman rank correlations for individual metals in relation to the biomass of individual groups of phytoplankton. It revealed a positive correlation between Chlorophyta biomass and concentrations of copper and lead (r = 0.98 and 0.91, respectively, *p* = 0.05). The Cyanobacteria and Bacillariophyta were positively correlated only with iron (r = 0.95 and 0.59, respectively), while the Cryptophyta correlated with barium (r = 0.69) and lead (r = 0.84). In spring, the heavy metals studied inhibited green algae, diatoms, CPOM accumulation, and total photosynthetic activity (PA). Their concentrations were positively correlated with cyanobacterial biomass (except for Fe, Mn, and Ni). In summer and autumn, Cr and Ni were the main activators of phytoplankton biomass growth, and they inhibited photosynthetic activity. In autumn, the presence of Bi and Zn had the same effect. In summer and autumn, Mn played an opposite role, i.e., it inhibited the growth of algae and increased their PA. This effect was additionally supported by Ba and Cu in autumn. Results of the correlation matrix (r) between the metal and chlorophyll-a concentrations throughout the seasons are given in Supplementary material (Table S3).

**Figure 3:**
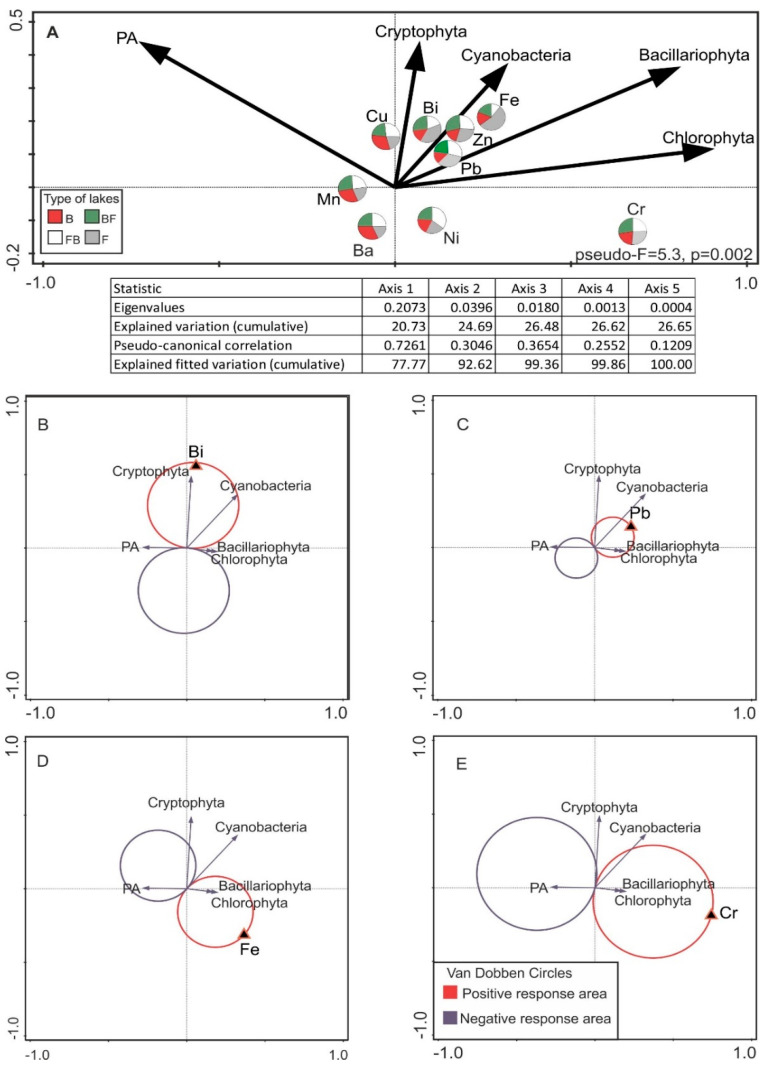
(**A**) A t-value biplot with van Dobben circles based on the redundancy analysis (RDA) of heavy metals in surface water and phytoplankton biomass (RDA, figure not shown); (**B**) the van Dobben circles for Bi; (**C**) the van Dobben circles for Pb; (**D**) the van Dobben circles for Fe; for Cr (**E**).

Relations between metal concentrations in lake water and phytoplankton structure were further investigated using RDA and van Dobben circles (Figure 3). The final model explained 25% of the total variation in the structure of phytoplankton, and all the canonical axes were significant (Monte Carlo test, *p* = 0.002). The first axis shows a gradient positively related to chromium concentration (which was similar in all the studied lakes) and negatively related to manganese, which was observed primarily in B (Figure 3A). The chlorophyll-a concentrations of green algae and diatoms were major contributors to positive values of the first axis, while photosynthetic activity was the most important factor for the second axis. In the plot of RDA ordination (axes 1 and 2), the metals that appear to influence phytoplankton structure can be distinguished. The RDA analyses clearly show the negative effects of Ba and Ni on the biomass of individual groups of phytoplankton, whereas Fe, Bi, Cr, and Pb seemed to stimulate them. However, on the basis of van Dobben circles and the spaces of positive and negative responses to predictors, we found that Bi was positively associated with Cryptophyta to a large extent and with Cyanobacteria to a lesser extent (Figure 3B). The biomass of Bacillariophyta and Chlorophyta seemed to be stimulated by the presence of Pb, Fe, and Cr (Figure 3C–E). Increases in Fe and Cr were associated with a decrease in algal photosynthesis.

## 4. Discussion

Most shallow-water coastal ecosystems are characterized by high habitat instability [31,32]. The variety of intensity of seawater intrusion into them is linked with temporal and spatial variation in marine currents and tides [11,33,34], which are principally driven by wind, so abiotic conditions change very unpredictably [35,36]. In our study, this attribute applies particularly to Lake Sarbsko (BF), which is relatively rarely recharged with seawater, and where phytoplankton biomass is much higher than in all the other lake types (Table 3). The change in freshwater status during seawater intrusion is brief because the intrusion is too small or too brief to result in a stable brackish phase. Such a situation is also more likely if a water body is oriented E–W, with its inlet and outlet located on that axis. This situation supports intensive mixing of fresh waters from the Chełst River and saltwater flowing from the west and from the Łeba River canal into the Chełst [37,38]. These dynamics increase the resuspension of sediments and the release of organic matter into open water, so that phytoplankton development is affected more strongly than in the other lake types [39].

The limited seawater intrusion does not favor a wide spatial dispersion of euryhaline species. In the four groups of phytoplankton we studied, the reduction of biomass in brackish lakes suggests that their open water is dominated by stenohaline organisms. In the ecosystems that are subject to seawater intrusion, the observed phytoplankton biomass was much lower than in transitional ones with a dominance of fresh waters or in typical freshwater lakes (Table 3). It is supposed that seawater intrusion causes osmotic stress in algal cells [40], except that Cyanobacteria seem to be more resistant to such changes so they are dominant in most lagoons and coastal lakes along the Baltic Sea, e.g., Cyanobacteria outcompete other groups thanks to their ease of adaptation to varying conditions and relatively fast growth [41,42], as confirmed by the results of our research.

In general, the low water quality in Baltic coastal lakes is caused by sewage discharge and intensive farming [43]. Trojanowski [44] showed that the catchment areas of Lakes Łebsko and Gardno are located in one of the most productive agricultural regions of Poland. The influx of pollution affects their productivity, which, in contrast to earlier reports, is low in these lakes. Their low productivity is unusual because the lakes are rich in phosphorus and nitrogen, but, according to Karlsson [45] visibility determines productivity. It is highly probable that the overproduction of Cyanobacteria, directly limiting visibility, simultaneously limits the development of other groups of phytoplankton.

Anthropogenic pollution, including by heavy metals, can cause structural changes and functional disturbances in bioseston. Of the organisms for which similar studies have been performed, phytoplankton commonly exhibit the highest concentration ratios for many elements, primarily due to their small size and corresponding large relative sorption area [46,47]. Investigation of the relations between algal biomass and heavy metals in the vertical profile is necessary to understand the structure and dynamics of changes taking place in open water. Phytoplankton are influenced not only by the concentration of a given substance but also by the broader chemical environment. Overall, the influence depends on concentration, exposure time, mode of metal uptake, internal factors, and environmental conditions. Many earlier studies have indicated that water pH indirectly affects the rate of metal accumulation. Salinity lowers the uptake and accumulation of metals, in contrast to temperature, which is positively correlated with their accumulation [48,49,50].

We found that heavy metals in the studied lakes seem to stimulate the growth of particular groups of phytoplankton (Figure 3). It is well known that there are very complex interdependencies between abiotic and biotic elements in aquatic ecosystems. Some of the relations are direct and others are indirect. Our study shows an indirect effect of chromium on phytoplankton development. The toxic effect of Cr on zooplankton has been proven [51], and this in turn contributes to increasing phytoplankton biomass due to reduced feeding. The effect of the metal increased the phytoplankton biomass while also decreasing their photosynthetic activity.

Another example is bismuth, which undergoes intense hydrolysis in its oxidation state III and is found in seawater as Bi(OH)_3_ [52]. Due to its extensive hydrolytic properties, Bi exhibits strong molecular reactivity and is therefore potentially toxic to marine species. Some elements, although normally toxic, can be favorable for some organisms in certain conditions. In spite of its toxicity, Bi apparently stimulates the growth of Cryptophyta and Cyanobacteria (Figure 3B) [53]. We have also found that even relatively small amounts of elements diminish the activity of metabolic processes of algal cells. An example is Ba, which, despite its low concentrations in the waters of the studied lakes, exerted a negative effect on phytoplankton growth (Figure 3).

Aquatic organisms vary in their ability to selectively accumulate an excess of metals. Lead is accumulated most strongly, and its degree of toxicity depends on the natural characteristics of species and the properties of water [54]. In this context, the metal seems much more dangerous to invertebrates than to algae [11]. However, in diatoms and green algae, it can be associated with an increase in photosynthetic activity, *sensu stricto* primary productivity (Figure 3C). Although Cu is quite widespread in nature, its concentration in unpolluted waters is low. It is subject to remarkable variation resulting from fluctuations in oxygen content because of algal production. Low concentrations of Cu salts limit the growth and photosynthetic activity of algae. However, algae can adapt relatively quickly to higher concentrations of this metal [55]. The toxicity of Ni is much lower than that of Cu. Chromium, despite its common occurrence, should be present at relatively low levels in the vertical profile. It is able to form oxyanions and can be oxidized to chromate [56]. In contrast, the reduction of chromate to Cr(III) is linked with free radical formation, which makes the metal highly toxic. Consequently, chromate is toxic, allergenic, and carcinogenic [56]. It is detoxified in bacteria not only by reduction but also by efflux [57]. In aqueous solutions, chromates are stable only in alkaline environments, such as the lakes investigated in this study. High Cr concentrations in water are associated exclusively with pollution. Undoubtedly, algal photosynthetic activity decreases when Cr and Fe concentrations are higher (Figure 3D,E), even though their concentrations fit within the highest water quality class, according to Polish environmental protection guidelines for transitional ecosystems [58]. In this context, Zn is an exception, as its values markedly exceeded the permissible limits (>1). Zinc has a negative effect on algae even at low concentrations and increases the toxicity of Cu. We presume that the observed phytoplankton community structure is conditioned by concentrations of these metals in water. Its solubility increases at low pH values [59]. Manganese is one of the most important heavy metals and is considered as an electron buffer for biochemical reactions. Its most conspicuous property is the ability to split water in photosystem II, which contains Mn in Cyanobacteria and chloroplasts. The system is responsible for nearly the entire mass of O_2_ in the atmosphere of Earth and for the oxygenation of Earth’s crust. The toxicity of Mn(II) is generally low, as compared with other transition metals [60]. The low Mn content of the studied lakes could be caused by Cyanobacteria uptake. In addition, free Mn ions can mitigate the toxic effects of Cu [61] or Zn [62].

Although the lakes we studied are highly polymictic (mean depth 1.4 m), heavy metal concentrations differed significantly between them. This observation confirms the importance of hydrological connectivity with the sea in shaping abiotic conditions in Baltic coastal lakes, e.g., [11,63]. Our study shows that two subtypes of Baltic transitional lakes (FB and BF types) can be distinguished, as they differ in proportions of fresh and seawater, although both are regarded as freshwater ecosystems according to the standard salinity classification [64]. This fact should be considered in further ecological analyses of polymictic coastal lakes with low salinity levels.

## 5. Conclusions

The study showed that phytoplankton communities in coastal lakes along the southern Baltic Sea undergo interactions with abiotic factors (water quality and hydrological connectivity), leading to changes in their structure and photo-synthetic activity. Contamination of water with heavy metals is an important factor that interrupts plankton organisms at the cellular level and potentially disturbs the ecological balance of the aquatic biota. Seawater intrusion is one of the abiotic factors that can restore a higher biodiversity of phytoplankton in coastal ecosystems. The influx of seawater negatively affects the typical freshwater species, but it also markedly limits the abundance of Cyanobacteria and the level of pollution with heavy metals. It is advisable to consider increasing the degree of seawater intrusion into brackish lakes to protect them against degradation. However, Cyanobacteria may then outcompete other groups (thanks to easy adaptation to varying conditions and fast growth), so it is also important to limit sewage discharge and intensive farming in the catchment areas of coastal lakes. Altogether, these actions would contribute to protecting these priority habitat types of Natura 2000, in line with the environmental policy of the European Union.

## Figures and Tables

**Table 1:** Morphological characteristics of the coastal lakes studied in a salinity gradient [7,22,23].

Lake Characteristics	Lake Łebsko	Lake Gardno	Lake Sarbsko	Lake Dołgie
Geographic coordinates	54°43′ N 17°25′ E	54°39′ N 17°07′ E	54°22′ N 18°48′ E	54°42′ N 17°12′ E
Area (ha)	7020	2261	611	136
Mean depth (m)	1.6	1.4	1.2	1.4
Max. depth (m)	4.7	2.2	3.2	2.7
Volume (10^6^ m^3^)	113.5	30.9	7.3	2.2
Salinity level (PSU)	1.5–7.05	0.3–2.9	<0.9	<0.02
Type of hydrological connectivity (sea connection)	Permanent seawater intrusion by the Łeba River	Periodical seawater intrusion by the Łupawa River	Occasional seawater intrusion by Chełst and Łeba Rivers	Fully isolated from sea
Residence time (days/year)	250 < x	150 < x < 250	0 < x < 150	x = 0
Type of habitat	Brackish (B)	Freshwater-brackish (FB)	Brackish-freshwater (BF)	Freshwater (F)

**Table 2:** Concentrations of heavy metals (mean value ± standard deviation) in the surface water of four types of coastal lakes (*n* = 156), and the results of one-way ANOVA evaluating variation between them.

		B *n* = 66		FB *n* = 30		BF *n* = 30		F *n* = 30		*p*
		Mean	±SD	Mean	±SD	Mean	±SD	Mean	±SD	
Ba	mg L^−1^	0.03	0.02	0.02	0.02	0.02	0.02	0.02	0.03	<0.0001
Bi	mg L^−1^	0.01	0.01	0.01	0.00	0.02	0.01	0.02	0.01	<0.0001
Cr	mg L^−1^	0.02	0.02	0.02	0.02	0.02	0.02	0.02	0.02	0.6
Cu	mg L^−1^	0.02	0.01	0.02	0.02	0.02	0.01	0.01	0.01	0.001
Mn	mg L^−1^	0.22	0.19	0.15	0.10	0.19	0.10	0.12	0.06	<0.0001
Fe	mg L^−1^	0.78	0.53	0.58	0.36	0.85	0.63	2.75	1.42	<0.0001
Ni	mg L^−1^	0.05	0.12	0.07	0.13	0.05	0.06	0.05	0.06	0.4
Pb	mg L^−1^	0.05	0.07	0.09	0.13	0.06	0.11	0.09	0.11	0.2
Zn	mg L^−1^	0.63	0.42	1.05	1.02	1.18	1.70	1.08	0.77	0.001
Total		1.78		2.01		2.41		4.10		<0.0001

*p* values modified by the Tukey procedure for multiple comparisons show no significant effect.

**Table 3:** Mean chlorophyll-a concentration (μg L^−1^ ± SD) of groups of phytoplankton and their photosynthetic activity (PA, %) in various types of Baltic coastal lakes and results of the one-way ANOVA evaluating variation between them. TChl = total chlorophyll-a concentration, PA = photosynthetic activity.

	B *n* = 66		FB *n* = 30		BF *n* = 30		F *n* = 30		*p*
	mean	SD	mean	SD	mean	SD	mean	SD	
TChl	13.73	13.76	18.59	17.45	98.17	183.81	55.32	82.88	<0.001
Chlorophyta	2.53	3.67	2.14	2.38	2.85	4.14	8.12	9.34	0.001
Cyanobacteria	10.28	9.66	15.94	15.77	80.27	158.17	43.20	71.65	0.001
Bacillariophyta	0.30	0.62	0.30	0.46	0.21	0.51	3.38	4.91	<0.001
Cryptophyta	0.05	0.17	0.02	0.13	14.64	27.74	0.46	1.17	<0.0001
PA	10.9	6.9	10.1	7.0	8.6	6.2	9.7	7.4	0.5

*p* values modified by the Tukey procedure for multiple comparisons show no significant effect.

## Data Availability

Not applicable.

## Supplementary Materials

The following supporting information can be downloaded at: https://www.mdpi.com/article/10.3390/ijerph19074131/s1, Table S1: Concentrations of heavy metals (mg L^−1^) in lake water, Table S2: Concentration of chlorophyll-a (μg L^−1^) and photosynthetic activity (%) in lake water, Table S3: Results on correlation matrix (r) between metal concentrations and chlorophyll-a concentrations in seasons.
